# Explosive and Harmful Impulses: A Subset of Child and Adolescent-to-Parent Violence and Abuse

**DOI:** 10.1177/08862605241244470

**Published:** 2024-04-09

**Authors:** Nikki Rutter

**Affiliations:** 1 Durham University, UK

**Keywords:** youth violence, anything related to domestic violence, domestic violence, vulnerability to abuse, developmentally delayed, DD offenders, developmentally delayed

## Abstract

“Filial harm” refers to harms experienced by a parent and caused by their child, with increasing umbrella terminology developing to capture all forms of harm despite differing experiences and contexts. In this paper, based on a Glaserian Grounded Theory study underpinned by participatory research principles, this work utilized diaries and interactive interviews with 34 parents and arts-based workshops with 21 children to develop a new terminology and approach to child and adolescent-to-parent violence and abuse when harm does not have a clear intent. Explosive and harmful impulses refer to preadolescents experiencing proactive, reactive, affective, and relational harms and needs. These specific forms of filial harm are based upon underlying needs, and the maladaptive ways children may attempt to meet their needs result in filial harm as an unintended consequence rather than being a form of harm with intent. Using an approach that captures subsections of filial harm, there is opportunity to better represent the nuance of individual family experiences and could provide more appropriate language and interventions that better represent the language used by families themselves. Future interventions, support pathways, and research with families living with explosive and harmful impulses could use the provided framework to understand why children are attempting to meet their needs in harmful ways and to consider less harmful methods of support.

## Introduction

Filial harm refers to harm experienced *by* an adult in a parenting role, instigated, or caused *by* their child (pre-adolescent, adolescent, or adult child). It has been of increasing interest to policymakers, practitioners, and researchers and is recognized as a distinct social phenomenon which challenges our existing understanding of parent-child interactions and dynamics. This distinction highlights the importance of continuing research and the development of appropriate interventions to support families living with this form of harm (Harbin & Madden, 1979; Holt & Lewis, 2021; Miles & Condry, 2015).

There are several names for filial harms which are instigated or caused by child(ren), formed due to political, social, cultural, and value-based differences between academics (Rutter, 2023): battered parents syndrome (Harbin & Madden, 1979); child-to-parent violence (Gelles, 1985); adolescent-to-parent violence (Cornell & Gelles, 1982); parent-directed aggression (Nock & Kadzin, 2002); child or adolescent-to-parent violence and abuse (CAPVA; Clarke, 2015); challenging childhood violent and aggressive behavior (Thorley & Coates, 2018); adolescent violence in the home (Kehoe et al., 2020); and the Canadian co-produced umbrella terminology “aggression toward family/caregivers in childhood and adolescence” (Gervais et al., 2022). A key issue in finding a name for this form of harm is the cause, purpose, and impact of such harm can be different depending upon various contexts in which it occurs. Therefore, in an attempt to cover *any* and *every* form of harm experienced by a parental figure instigated by their child, be they preadolescent, adolescent, or adult child, the language evolves and is debated.

The aim of this article is to focus on how we (the researcher and co-researcher participants) engaged in an iterative methodological process to refine and define terminology which reflected co-researcher experiences more authentically than the language and terminology currently being utilized within filial harm research, practice, and policy. In this paper, I argue the increase in umbrella terminology to capture every form of filial harm is becoming increasingly unhelpful to both researchers and practitioners, and instead, there is potential to capture the nuance of these experiences by naming sub-divisions of such harms. These specific forms of harm are conceptualized within what we term a ‘PRAR’ needs-based framework, which highlights similar presentations of filial harms that may be underpinned by a different needs-basis; thus, different interventions and approaches may be required. PRAR stands for **p**roactive (goal-directed behaviors whereby harms achieved a specific goal or outcome), **r**eactive (responding to harmful stimuli), **a**ffective (intellectual, physical, social, and/or emotional), and **r**elational (associated with attachment, intimacy, closeness, belonging, connection) harms and needs.

These more specific forms of filial harms may require more specific interventions and support systems, as they are based upon underlying needs and the maladaptive ways children and young people may attempt to meet these needs (Harries et al., 2024). By using an approach that captures subsections of filial harm, there is opportunity to better represent the nuance of individual family experiences, whereas the umbrella terminology can be used when attempting to capture the broader experiences relevant when making wider policy changes.

### Harm and Vulnerability

As much of the current literature around filial harm is based upon small sample sizes within therapeutic settings (Harbin & Madden, 1979; Micucci, 1995), policing or criminal justice data where families have hit crisis (Holt & Lewis, 2021; Miles & Condry, 2016), or community samples which take an overly inclusive view of filial harm (Jaureguizar et al., 2013), such evidence has been referred to as the “tip of the iceberg” (Miles & Condry, 2015, p. 76). This means those families who seek support from services for filial harm are often those who are already experiencing multiple compounding factors, which reduce their capacity to manage the filial harm or the filial harm is escalating. They are already in contact with various support services; thus, they may be more visible in filial harm samples. For instance, children and young people from care-experienced backgrounds who are neurodivergent and/or have specialist educational needs appear to be more likely to be vulnerable to instigating this form of harm (Coogan, 2017). These populations are also more likely to struggle with emotional regulation, be hypervigilant, reactive, or avoidant to uncomfortable challenging stimuli, and have difficulty with creating or maintaining positive relationships (Gabel et al., 1994). The challenges facing children instigating filial harm alongside their care experience, neurodivergence, or specialist educational needs all increase internalized and external stigma (Goffman, 1959), which compounds the emotional, social, and relational challenges which could, in turn, escalate the frequency and intensity of relational harm (Coogan, 2017). Furthermore, these compounding factors disempower children and young people who are concurrently instigating and experiencing harm, and thus, it is important the language used to describe this harm does not further stigmatize them.

### Control and Intent

A key issue when using language associated with violence, abuse, and aggression when referring to child-instigated harms is it implies intent, as much of the existing violence and abuse discourse refers to power, control, and intent, and so these framings are then applied to children and adolescents instigating harms (Molla-Esparza & Aroca-Montolío, 2018). This becomes a particular problem when evidence suggests many harms instigated by children and adolescents within the home are not directly intentional but rather expressions of overwhelming distress, a symptom of a wider issue, or an unintended consequence of trying to meet their individual needs (Rutter, 2021, 2022; Thorley & Coates, 2018).

Much practice and policy frames filial harms instigated by adolescents as a form of domestic abuse, which reproduces conflation between existing domestic abuse literature and filial harms (Bettinson & Quinlan, 2020; Clarke, 2015; Holt & Lewis, 2021; Home Office, 2021). This may be due to the similarity in experiences, as parents have previously referred to the experience of *walking on eggshells* when living with harm, which resembles adult experiences of partner abuse (Routt & Anderson, 2014). Coercive control is a subtype and fundamental component of domestic abuse, which can exist without any other form of domestic abuse, but all domestic abuse has an element of coercive control (Stark, 2007; Stark & Hester, 2019). Nevertheless, Bettinson & Quinlan’s (2020) study into whether “adolescent-to-parent violence” met the threshold for coercive control using practitioner focus groups found significant overlaps between these experiences; however, the vulnerability of both adolescents and parents meant the phenomenon had fundamental differences to adult instigated coercively controlling behavior. They recommended different responses that responded sensitively to these vulnerabilities.

These fundamental differences between filial harm and domestic abuse have resulted in debates regarding how to understand and conceptualize this phenomenon when the harm caused does not appear to be intentional due to the developmental or chronological age of the child, their neurodivergence, or mental health needs (Coates, 2018; Coogan, 2017; Thorley & Coates, 2018). There has been an emphasis that children instigating harms should be recognized as children first, and their chronological or developmental stage should be considered when applying models which were created and tested for adult perpetrators of domestic abuse (Condry & Miles, 2021). Within filial harm research and practice, there has been significantly more effort dedicated to applying models and approaches designed for adults than the development of grounded approaches which centralize the voices and experiences of families living with filial harm instigated by children (Rutter, 2022).

## Methods

This article is based on a larger Economic Social Research Council-funded doctoral research project exploring filial harms instigated by pre-adolescents whereby I utilized a Glaserian Grounded Theory methodology underpinned by participatory principles to be grounded in and centralize the voices and experiences of children instigating filial harm, and parents experiencing these harms, rather than applying preconceived notions relating to family violence research. The Glaserian Grounded Theory approach provides research questions, including what is the main concern of the substantive population (in this case, what conceptual language and terminology is considered appropriate by children and parents living with filial harm)? And how is this concern resolved or processed? (Glaser, 1998; Lazenbatt & Elliott, 2005).

Glaserian Grounded Theory requires openness, theoretical sensitivity, and conceptual clarity whereby analysis occurs through inductive, deductive, and abductive engagement with the data and is utilized to constantly compare generated concepts and conceptually categorize them through memoing (Glaser, 1978, 1998, 2021). Consistently referring to the research questions for clarification and to ensure data boundaries. As my Grounded Theory approach was underpinned by participatory principles, I engaged in a co-researcher approach to the work, whereby parents and children were considered research assistants (hence, co-researchers), and myself the primary investigator to ensure theoretically sensitive consistency throughout the research (Glaser, 1978). Through this approach, I considered co-researchers to be the experts in their own lives, families, and experiences. This research was undertaken in the period July 2020 to July 2021, during COVID-19, whereby there were intermittent lockdowns in locations around the globe in response to the pandemic, this impacted both the recruitment and the methods.

### Recruitment

Unrelated parents and children were involved in the research. Parents were recruited in two separate phases: July 2020 and September 2020. In the first round, I opted to recruit parents of children aged 4 to 11 years old through open sampling via two online support groups available through Facebook, requesting parents were experiencing “conflict” with their child. While social media users are not considered to be representative of the general population, often younger, with have higher-level qualifications (Mellon & Prosser, 2017), these groups were ones I had affiliation with through “real-world” interactions. This first round resulted in interest from a self-selecting sample of nine potential parent co-researchers, and this was followed up with an email explaining the basis for the research and phone call or video call to explain the research project purpose, process, and stages. Parents were informed the research would be grounded in their priorities (in line with Glaserian Grounded Theory principles). It was emphasized they are the experts in their lives; we would be co-researchers.

The second round of recruitment occurred 12 weeks later, whereby parents had independently identified the more demands their child experienced, the more intense or frequent the filial harm. There was also a significant amount of neurodivergence with the children (autism, Attention deficit hyperactivity disorder (ADHD)), and one parent had begun a private assessment for “pathological demand avoidance” (PDA) for their child. PDA has been defined as a form of neurodivergence related to an autistic profile and is characterized by “an obsessional avoidance of the ordinary demands of life coupled with a degree of sociability that allowed social manipulation as a major skill” (Newson et al., 2003, p. 596).

Parents wanted to recruit others to the research who had identified that demands produced filial harm prior to starting the research, so I contacted a charity dedicated to PDA. They supported the work by sharing a poster on their social media pages. Eighty-four potential co-researchers responded to the advert, and I repeated the recruitment process above. Of the 84 interested individuals, 26 were recruited based on age of their child, whether they would be able to engage with the methods, and whether their child’s behaviors were appropriate to the research topic. In total, 34 parents agreed to be co-researchers.

From June 2021 to July 2021, 21 children aged 7 to 11 years old, unrelated to parent co-researchers, took part in weekly arts-based participatory workshops within a specialist primary school for children with social, emotional, and mental health needs. Two children from each class group were recruited into the research and were identified by parents and teachers as experiencing poor emotional regulation and awareness and instigating filial harm within the home.

### Parent Methods

As many parents were in lockdown, home-schooling their child(ren) during this research, methods which could mediate the potential presence of children needed to be considered. Furthermore, as this was a Grounded Theory study, I was particularly interested in the everyday interactions whilst working with parents of pre-adolescents in a way which can “make visible the whole person,” such as diaries, rather than a snapshot of their existence, such as interviews (Bartlett, 2012, p. 1717). Thus, diaries were the main form of data generation, with interactive interviews used to explore and analyze data with parent co-researchers.

Diaries are particularly useful in recognizing individuals can hold contradictory, complex, and overlapping emotions, and diaries have previously been utilized to evidence the challenges associated with parent-child conflict (LoBraico et al., 2020). Diaries were iterative, open, and selective, providing space to reflect but also providing opportunity to “follow the data,” which is a requirement of a Glaserian Grounded Theory approach (Glaser, 1978, 2021). Interactive interviews then took place every 6 weeks to provide updates and a platform for discussion and co-analysis between myself and each individual parent. This was an opportunity to analyze, direct, and sample data further. Interactive interviews, particularly when used in online contexts, have been defined as an ethnographic process effective at assisting in relationship-building with participants and involve numerous meaningful conversations about the topic of interest (Crichton & Kinash, 2003). It was at these interactive interviews, in conjunction with reflections found within diaries, that discussion and debate around names and definitions occurred.

Parents were initially asked to journal or reflect on their lives, families, and experiences in whichever way they felt most authentic. I define “diaries” in a very broad sense, as some parents did not have regular access to the internet; I accepted whatever method was most useful to them and their lives. Therefore, submissions included WhatsApp voice notes, emails, photographs of written diaries, video diaries, cloud-based storage links to online diaries, general reflections, daily recordings, or weekly overviews, as parents used all of these to record their lives and experiences. Interactive interviews were held either online or via phone, and as every aspect of the research was voluntary, not every parent engaged in every interview. Many of the diary entries did not focus upon incidents of harm but were descriptions of events that provoked difficult emotions in the parent, and thus, the diaries were used as a process of catharsis, although this was not the case for everyone. Parents also directed me to blogs, books, conferences, speakers, and YouTube videos they thought could be relevant to the research and recommended I access or attend these when they felt the specific sources reflected their own experiences.

Parents were involved in analysis throughout the process. I conducted the first part of analysis through identifying concepts utilizing constant comparison, returning these concepts and memos to parents for them to further consider at our interactive. This promoted depth of inquiry as they engaged with further constant comparison with their lived experience during these interviews in a collaborative way. Through these processes, I worked alongside parents as collaborators, going through their data line-by-line to identify concepts, constantly comparing the concepts to build categories, and memoing throughout.

### Child Methods

The school the work took part in identifying a space within their grounds which would only be accessed during the period of the research by myself, the children, and learning support assistants who would support the research. They also provided financial and practical support and invested in supplies for the children, which included small rewards to thank them for their participation each week. Children as co-researchers participated in weekly creative workshops in groups of four or five, with each workshop lasting 45 min. In each workshop, the children directed the data generation, choosing a difficult emotion, and the creative processes (art, dance, movement, games) to explore how these emotions could create harmful behavioral expressions in their home and school environment. Creating art can be a cathartic way to explore challenging and evocative experiences, such as violence, as well as providing an approach that facilitates children’s roles as co-researchers, as they not only generated data but designed and implemented the methods (Bird, 2018; Brady & Brown, 2013).

The workshops were all recorded, and the work and discussions were utilized as data, as were field notes I undertook throughout each workshop. Children would use space for discussion to analyze their own and one another’s artwork, constantly comparing throughout. To integrate this data into the wider dataset, which included the parents’ data, I constantly compared the concepts between the two data sets (parents and children) to check the relevance and appropriateness of the categories, memoing throughout.

### Ethical Considerations

The Economic Social Research Council (ESRC) framework for research ethics, and University guidelines were adhered to, and informed consent was given by parents and assent by children. Children and parents were unrelated to reduce the risks that may have occurred if they were to compare their experiences of filial harm beyond the research environment, using the research process as a catalyst for further harm. Parents were offered anonymity, with opportunity to be named if they wished, and the co-researcher, co-productive element of this work enhanced it ethical approach, as it was underpinned by relational practice and an ethics of care whereby the needs of the co-researchers were prioritized over the needs of the research (Banks & Brydon-Miller, 2018; Dominelli, 1998). Parents who did not wish to be identifiable have pseudonyms, as have all children. In a previous piece of research into “child-to-parent violence,” I found participants were sharing the artwork they had created on open social media accounts and explaining what it was for (Rutter, 2021). I recognize that, for some parents living with these forms of harm, sharing experience with others can be empowering.

I attempted to ensure all co-researchers had full and direct participation in this research; in line with promoting the dignity, rights, and welfare of research participants (Dominelli, 1998). Data was stored on a password-protected laptop, and transcribed Word documents were all password-protected. As consent is a process, I continually reviewed the ethics of the work, and when co-researchers no longer attended sessions or stopped replying to emails, I assumed they had withdrawn consent. Data generated by any co-researcher prior to this was still included, and no one requested their data be removed from the research pool. When children withdrew assent, this data was not included in the data set (for further detail, see Bennion & Rutter, 2024).

## Illustrations

In this section, I present illustrations that respond to the research questions: what conceptual language and terminology is considered appropriate by children and parents living with filial harm? And how is this concern resolved or processed? For the question relating to language, “the explosive child” fit both a concept and description that was considered appropriate by those living with filial harm instigated by children aged 4 to 11 years.

### The Explosive Child

As parents directed me to blogs, books, conferences, speakers, and YouTube videos they considered represented their experiences, there were some consistencies, including the book “The Explosive Child” (Greene, 1998). No parent was comfortable with the language of “child-to-parent violence,” which was terminology I had used in previous work and was what I believed was the most used terminology at the time of the research (Rutter, 2021). It became clear in the early interactive interviews that all first-round parents disliked the language of violence, and this was true of almost all second-round parents, too. Nevertheless, all but one of the parents confirmed they did recognize the behavior as violent, or violence. The framing of “child-to-parent violence” or CAPVA was deemed provocative, and parents did not want to describe their relationship with their child using the language of violence or abuse despite acknowledging the harm was being caused.

Every time I read “violence” in your research it makes me feel. . . just uncomfortable, even though I know what we have with “Amy.” It is violence. (Josie)

It is abuse, I am abused and it’s taken me a long time to get my head around that. But it’s his crocodile brain causing it. He’s not abusive. (Lou)

Parents all wanted better terminology for their experiences, which did not imply intent or blame, and whilst “violence” and “abuse” were rejected by parents, these terms were not used by children at all. Nevertheless, the “explosive” component used by Greene (1998) did represent their experiences, with children also using descriptions of explosions to describe some of their harmful behavior “I just go, like, boom!” (Tony, 7 years old); “like a bomb” (Scout, 8 years old). Therefore, we agreed new terminology should capture this explosiveness. Almost all the parents had read “The Explosive Child” (Greene, 1998), and recommended I included it in the wider research. Those who had not read it, had heard of it, and intended to read it at some point themselves. The title attracted parents because it described their experiences and the experiences of their child; for some, it was the unpredictability of the harm; for others, it was the significant destruction these harms could cause; for others, it was both. However, “explosive” did not capture every and all harm experienced and developing a more comprehensive name for these experiences was an iterative process, and not one which was immediately resolved.

Once the “explosive” description of the behavior was accepted by all the parents and was relevant to almost all children, it needed to be extended. As mentioned, not all behaviors were explosive or felt like “a bomb,” or an explosion of emotion; some were about managing the environment and other people within it. For some families, this component was the most significant in their lives, as they felt they were being “micro-managed by a child” (“Izzy”). So “explosive behaviors” was extended to “explosive and controlling behaviors,” despite some of the issues mentioned earlier regarding some of the problems of conflating filial harm with coercive control mentioned earlier. However, at interview, it became clear “explosive and controlling behaviors” did not meet the needs of approximately half of the parents, with one explaining:

Even explosive and controlling behaviours. . . like, I dunno, it gives me that tightening in my chest, I don’t like it. It’s the behaviour part. . . like it’s a choice. School say behavioural when they don’t want to give support. CAMHS call it behavioural when they want to say it’s my parenting, or him making a choice. It’s not a behaviour, it’s not a choice, it’s an instinct or something. We need a word that isn’t behavioural, and isn’t violence but shows it’s explosive and controlling but not behavioural. (Jessica)

Similarly, another parent avoided language they considered blaming the child and wanted terminology that avoided language which could be interpreted as blaming. However, one parent explained how she would avoid this type of language when speaking about her child publicly but would use it when seeking support, advice, or intervention:

Explosive and controlling behaviour applies both then and now. I don’t like to think of [my son]’s behaviour as controlling, and don’t describe him as this to anyone (with the exception of doctors), as if you say it out loud I think it’s like the parent placing blame on their child (I think that’s why I had the initial gut punch reaction I did on our zoom). However, it would grab my attention and wouldn’t stop me accessing the content. (Katie)

As the parents wanted to be clear, they did not consider their child’s behavior to be about them being a “bad” person, “behavior” had negative connotations. Reports from parents indicated when they first initiated help-seeking behaviors, frontline services termed the challenges the families were facing as “behavioral,” and used this as an indicator of why there was no need for intervention. There was an emphasis on this language as being overtly blaming, and they wanted to move away from any connotations of blame. Some parents also described this framing as why some practitioners interpreted the behavior as evidence the child was “bad” or “naughty” or the parent was “negligent” (Nicola). Parents further explained they had inferred from these responses this was services implying the behavior was deliberate or the parenting was to blame. Thus “explosive and controlling behavior” needed to be altered to remove the behavioral element and emphasize it was an instinctive response of impulse; thus, we developed the terminology “explosive and controlling impulses.”

One key development from the perspectives of parents in this research was they wanted evidence that being explosive and controlling was necessary for their child(ren). They understood the harmful behavior as being underpinned by a need, and the harm itself was unintentional but a consequence of harmful methods of attempting to meet those needs.

The terminology of explosive and controlling impulses appeared to be a more descriptive and identifiable name for families experiencing and managing such harms. It was considered less judgmental, less stigmatizing, and was co-produced with both parents and children. These impulses do not involve “intent” in the way intent is understood in the broader family violence literature. They were not considered a calculated effort to control or manipulate a parent. However, we changed *explosive and controlling impulses* to *explosive and harmful impulses* for reasons I will discuss later.

### Explosive and Harmful Impulses

The Grounded Theory developed in this research is what I term a “PRAR” needs base, which reflects both the underlying need and the harmful impulses children experienced in trying to meet that need. The four different types of harmful impulses that sit within the “PRAR” framework are: proactive, reactive, affective, and relational, which I will now cover.

#### Explosive and Proactive Impulses

This category represents goal-directed behaviors, whereby harms achieved a specific goal or outcome. This did not necessarily mean it was an intentional or pre-meditated behavior, but rather it was often a consequence of the child wanting something or wanting to avoid something in the future. The impulses and behaviors that fit within the proactive category were often used to help a child feel in control of the environment and helped them avoid what they may otherwise consider unpleasant or unsafe (i.e., transitioning to a new activity when in flow). There will usually be an identifiable outcome to the behavior, for example, a parent tells child they can’t go on a game, so child is destructive until the parent relents or a child wants to avoid going to school, so they hit and kick to avoid being made to put on their uniform.

Related to the above, anxiety was of relevance to this category, which is reflected in the second-round recruitment whereby first-round parents identified demands for increased incidents of harms and identified their child was highly anxious, and this anxiety increased the frequency and intensity of explosive and harmful impulses when there was the potential for demands to be made of the child. One demand that was difficult for parents to navigate was poor sleep. As a concept, sleep had several different characteristics which meant poor sleep for the parent rather than the child. Some children required medication to help them sleep, which could be anything from a low dose of melatonin (a hormone that is naturally produced to help us feel drowsy, but some individuals are not receptive to it or do not make enough of it [Parker et al., 2019]), to sleeping tablets. Some children would present with more explosive and harmful impulses at bedtime than at any other time. Poor sleep often resulted in co-sleeping, and whilst this could be the child in bed with a parent, it could sometimes mean parents sleeping on the floor of their child’s bedroom. In one example provided by a child, her elder sister would sleep on the sofa in their living room, despite having a bedroom; “she does it for me because I need a lot of space at bedtime and when I get up through the night, and she does it to help Mum because she is older” (Leanne, 9 years old). Alternatively, poor sleep could refer to parents barricading themselves and other children in a bedroom throughout the night to avoid being directly harmed by explosive and harmful impulses. This was more likely with children who were being proactive in attempting to have their immediate needs met.

As the proactive category was those explosive and harmful impulses that were engaged in as a method of avoiding something unpleasant or undesirable, it was often presented as parents avoiding saying or doing something they knew would cause an explosive or harmful impulse or walking on eggshells around their child. Sometimes, however, an undesirable event was unavoidable, and children would present with explosive or harmful impulses in an attempt to prevent the event from happening, as was found by “Hannah”:

[H]e sat on the floor by the door refusing to put his shoes back on and seeming increasingly agitated. As we were blocking the entrance, I was feeling pressure to get moving, so I offered “Josh” the choice of putting his own shoes on so we could leave, or I could do it for him. He still refused, so I leaned down to help put them on and he became immediately aggressive towards me, kicking out and kicking my arms hard repeatedly as I tried to get his feet in his shoes. (Hannah)

School avoidance was the most recorded proactive strategy, but many parents recorded different proactive explosive and harmful impulses, and proactive needs were found even in those children who did not appear to engage in explosive or harmful impulses for reactive needs. School avoidance has been recognized since the 1940s (Klein, 1945), when there was a focus on anticipatory action. This school avoidance could be grouped into three different motives: anxiety, aggression, and secondary gains. In this research, parents were not separating school avoidance into three motives, but instead, the three motives were symptoms of the avoidance. Below is an example given by a parent of how their child used different explosive and harmful impulses to prevent his parents from sending him to school.

He was refusing to go to school, wouldn’t get his shoes on was thumping doors and picking things up to throw, went and sat in the garden screaming that school is boring. (Kalley)

Proactive behaviors often resulted in parents changing their own behaviors to pre-empt explosive and harmful impulses, often unsuccessfully. Thus, we found they also started to engage proactively with their child to avoid the harms. Furthermore, there were many examples of children attempting to regulate or calm themselves proactively through sensory-based strategies, more of which will be explored in the “affective category.”

#### Explosive and Reactive Impulses

This category identifies needs which are attempted to be met through a reactive response and can be observed in three separate contexts. Firstly, the reactive category can be observed *in response* to explosive and harmful impulses (usually via the parent), whereby a harmful impulse occurs, and there is a reaction to this to meet the needs of that family member. A second example of the reactive category can be a family member reacting to something negative *to prevent* an explosive and harmful impulse (usually the parent, but occasionally the child), which can be a strategy to avoid escalation of distress. Finally, the reactive category can be understood as *causation*, whereby an explosive and harmful impulse occurs as a reaction to unpleasant or undesirable stimuli (usually the child).

Some reactive responses are understood through physiological responses, such as fight or flight as different responses to the same or similar stimuli. Responses can be immediate or delayed, and this is dependent upon the stressor and the individual. For instance, children who mask their needs in education, were more likely to have delayed reactions. Masking is a behavior in which an individual consciously or unconsciously changes their behavior to avoid stigma in social environments; however, masking has been associated with poor outcomes for the masker, who may struggle with emotional regulation and mental illness in the long term (Miller et al., 2021).

Challenges with school were not wholly navigated proactively. For some parents, education fit within the reactive category and consisted of concepts such as masking, workload stress, and friendship issues. In the reactive category, education frequently resulted in the coke bottle effect, whereby children gradually became more “shaken up” (distressed) throughout the day but waited until they were picked up from school or until they arrived home to “explode,” that is, react to their stressors. This was compounded when education provided a high level of strain on a child, whereby they experienced and were constrained by adult expectations, that is, the expectations placed upon them by teachers, learning support assistants, and the broader social expectations that impact children as they grow and learn. Thus, some children contained their emotions until they were in an environment where they were less constrained by social expectations (i.e., the public sphere) and could “explode” away from them in the home (i.e., the private sphere). Some parents and previous research described this as the parent being “the soft, warm comfort that lets [a child] know he is safe” (Rutter, 2021, p. 1327), and this feeling of safety was framed as the reason why explosive and harmful impulses could occurred within the home but not outside of the home.

Schools provided routines and routines can be helpful; school can also provide space between parents and children when they need space from one another; schools can also be unpredictable, with changing staff and pupil populations, and occasionally, appointments may mean a late start or early pick up is required, which can result in explosive and reactive impulses:

“Josh” became distressed and violent in the car. He screamed loudly and was kicking the backs of the other chairs and generally very upset to the point we couldn’t reason with him. We got home and he went up to his room and trashed it, throwing everything around and banging against walls. He also snapped his TV remote by throwing/banging it against the wall. I think the trigger for this was suddenly taking him from school before home time. (Hannah)

While the above reactive impulse appears to be related to distress “Josh” experienced at having his routine disrupted, other explosive and reactive impulses can be reactions to a disruption in activities that are pleasurable, enjoyable, and/or planned. For instance, Michelle has learned not to interrupt these types of activity, as it will result in an explosive and reactive impulse:

When she came home from school today and announced she was making a surprise I could have said no but it is just not worth the upset it would have caused [proactive response]. If [my daughter] has a plan in mind, she really struggles to deviate from it and saying no can result in lots of crying, shouting, swearing, verbal abuse and sometimes physical abuse too from [my daughter]. Put simply-aggression. (Michelle)

Similarly, “Hannah” experienced explosive and reactive impulses from her son when his plans were disrupted or he was unable to access activities that brought him pleasure, such as his tablet:

“Josh” asked for his tablet to play games and I said this wasn’t charged yet. He immediately became very aggressive. He threw hard heavy toys across the room towards me and his sisters and kicked my 2 year old so I had to move her across the room out of his way. He then tried to get to his sisters to kick them and I blocked him from moving. “Josh” then started screaming hysterically and attacked me, kicking hard, punching and I was unable to fully control the situation due to his size. I shouted for my partner. . . to come and help, and he picked “Josh” up to take him upstairs to his room where his sensory area/toys are to calm down. He screamed and thrashed around while being carried up the stairs and repeatedly kicked and hit [my partner]. (Hannah)

This was supported by children who consistently reported computer games and activities, which were typically considered reliable and important, would create a reactive impulse when the activity did not go as expected:

I was just playing, and then it just happened, and then I was banging my head off the wall over and over really hard, just rage. . . I was, like, trying my best to not get angry, but it was really hard, and I did. (Malcolm, 6 years old)

#### Explosive and Affective Impulses

The affective category refers to those needs which are met through intellectual, physical, social, and/or emotional stimulation. Much of the category relates to sensory needs, as parents frequently reported their children had challenges with proprioception (an awareness of where their bodies are positioned in space); experienced hyper-sensitivity (an over-reactive sensitivity to stimulation), which could result in pain, unexpected discomfort, and what could be perceived as an over-reaction; or hypo-sensitivity (an under-reactive sensitivity to stimulation), which could result in poor awareness of how rough they were being with others, how hard they had hit, or would promote sensory-seeking behaviors whereby there was an urge or an impulse to engage in harmful acts (Gabel et al., 1994).

As most parents identified their children were neurodivergent, this was frequently presented as challenges relating to discomfort of difficult sensory experiences, which meant many explosive and harmful impulses were triggered by everyday expectations. Bathing, hair-brushing, and certain clothing became distressing for the children living with touch hyper-sensitivity, and their need to avoid this resulted in proactive or reactive responses.

However, those children with touch hypo-sensitivity were attempting to meet their needs through physical aggression. In many cases, hurting other members of the family was physically enjoyable to the child as they met their affective needs through harming others. In some cases, this was navigated by family members who encouraged and promoted alternative activities, such as body socks, trampolining, or physical play. Nevertheless, as many of the children with explosive and harmful impulses struggled to self-regulate, often the activities would escalate until the child or their siblings became distressed, and when a child tried to avoid hurting others, they would hurt themselves instead. This self-harm could involve biting, scratching, head-banging, or destruction of their own property.

Bedtimes were a trigger for explosive and affective impulses as well as explosive and proactive impulses, according to many diary records, with some children requiring melatonin as a sleep prompt, and others reported their children required significant sensory stimulation before they were ready to sleep. In the case of Josie, her daughter “Amy” began taking melatonin during the research, which made a significant difference to what had previously been a “fraught” bedtime routine:

Bedtimes are often really fraught in our house and tonight was no exception. [my husband] had to ask her repeatedly to brush her teeth and he ended up doing them for her which she hates. Then she was messing about pulling all the covers off our bed and wearing the super king duvet as a cloak. She then tripped on it and banged her head on the skirting board and cried. This sort of thing happens a lot at bedtime, however calm we try to keep it. . . Eventually she calmed down and went to sleep about 9 pm after making a nest of all her cushions and blankets on her bottom bunk. Come to think of it, making a nest of cushions is a sure sign that she’s stressed or anxious. (Josie)

Thirty parents reported their child had issues around food and eating, some noticed an increase in explosive and harmful impulses when their child was hungry or thirsty and so they managed this proactively by “always send[ing] him in [to school] with extra drinks and snacks, to make sure there is no hunger or thirst issues” (Emma). Whereas others found their child had a very restrictive diet and a restrictive way of eating. This was an additional burden on parents, as they had to include additional tasks in their day to accommodate these dietary restrictions. Similarly, “Scout” reported when he experienced explosive and harmful impulses, he also experienced “angry poos” whereby his physiological responses extended beyond explosive and harmful impulses, and eight other children reported the same experiences.

In one diary example whereby “Jessica” reflected on when she would see increased frequency or intensity of explosive and harmful impulses, she explained how she noticed explosive and affective impulses could emerge from enjoyable activities due to her son finding it difficult to regulate or calm himself. Thus, these impulses escalated until he was overwhelmed:

A pattern of behaviour I have not taken much notice of became really apparent this week. [my son] seems to run an emotional rollercoaster (I am aware of this but I haven’t really acknowledged the sequence). [my son] has been playing with his sister a lot. Between them they get very giddy; running around shouting, giggling and making up silly words. [my daughter] is able to bring herself down without major incident. [my son]’s mood elevates to a point at which it is hard for him to come down. This means that they only route he has to bring his mood down is by becoming very upset. (Jessica)

Thirty parents indicated bolting behaviors, which involve a child running away from their caregiver, were common as soon as their child could run. This was frequently described as unexpected, and their child was indifferent as to whether they were being followed or not. Despite bolting being a common feature of children with developmental differences, multiple parents explained they had been told variations on “they’ll come back when they realize you aren’t following them,” rather than being advised these behaviors were indicative of neurodivergence (Call et al., 2019). In every example, parents explained their child would not come back; bolting was not about reacting to something negative or a method of seeking attention, but the need to run.

#### Explosive and Relational Impulses

A relational impulse is one which is connected to other people. It, therefore, involves attachment, intimacy, closeness, belonging, and connection but also attention and adoration. At the other end of the spectrum, there was also loneliness and isolation. The consequence of this was particularly seen in children who experienced rejection sensitivity and in those children who are often seeking connection with others. Conflict in the relational category occurred when both parent and child were seeking to have their relational needs met in different ways.

At the individual level, children who presented with explosive and relational impulses were described by their parents as having very low self-esteem, although this often appeared to look like overconfidence. This was also reported by teachers of the child co-researchers. Children who experienced explosive and relational impulses benefited from one-on-one time with adults and managed their impulses better when they had undivided attention. Children who presented this way have historically been pathologized in terms of classifying them with a disorganized attachment style or personality disorders (Fonagy, 1999), as their need to connect and feel connected with others is significant.

Parents identified many relationships which were helpful or not so helpful when their children had explosive and harmful impulses, and this often related to relational needs but could increase the frequency of explosive and relational impulses. For instance, in the case of “Catherine,” she found receiving support from family members and the opportunity to have space from explosive and harmful impulses was important, but family members could also increase her feelings of isolation when the family members who recognized support was required were less skilled in navigating explosive and relational impulses:

I think my sister, as helpful as she is at times, is a big trigger for [my son]. He picks up on her frustration and uses that. A couple of times she has been sharp with him which has triggered him. And it hurts me to say I think I’d be better without her interference (not sometimes as sometimes I’m at the end of my rope with everything an need someone there so I can take five) but it’s left me feeling more lonely. (Catherine)

Most parents reported their relationship with their children varied day to day. Some reported it was mostly positive other than when there were incidents of explosive and harmful impulses; others reported it was mostly negative with the occasional positive. When the explosive and harmful impulses were as uncontrollable as they were with some of the children described by parents, some children appear to have identified specific members of the family to be the target of the ECI, such as “Josh,” who was in a family of six, but the majority of explosive and harmful impulses were experienced by his mum or his older sister:

[His] older sister . . . tried to speak to him, he suddenly lashed out. He chased after her causing her to run as she was scared he would hurt her, he then caught up to her and started attacking her—punching and kicking until she was in tears. I caught up and pulled him away, he started to hit me and ran off. (Hannah)

In the above example, “Josh” would direct his explosive and harmful impulses toward the two people “Hannah” reported to be the most sensitive to his distress. As to whether this was an example of sabotaging those relationships is unclear. As rejection sensitivity can be understood as a relational need, many parents provided evidence of their children presenting in a way that could be conceptualized as rejection sensitivity. For instance, several parents described the difficulties their child had when a sibling had a friend at the house, such as the case of “Harriet”:

If [my daughter]’s older sister has a friend round then [my younger daughter] can be quite manipulative and will encourage her sister’s friend to come and play with her instead—which understandably upsets her sister. (Harriet)

In a different example, Emma described how her son needed to feel included and was very sensitive to rejection, shame, and embarrassment. This meant Emma had to work with school to avoid any of these feelings, or there would be explosive and harmful impulses: “He gets really upset if he can’t walk home with his friends. And he gets really embarrassed if his teacher has to talk to me” (Emma).

The explosive and relational impulses parents reported were often presented in a direct way; however, there were indirect challenges, too; some explosive and harmful impulses were not observed until well after the event, such as the case of Josie, who had a number of relational needs not being met, and her daughter who was trying to navigate feelings that sometimes resulted in causing harm:

So this morning “Amy” tore up my Valentine’s card from [my husband] (just after I’d spent some time digging out an envelope, stamp and [her friend’s] address so she could send one to [her friend]). She denied she’d done it but it just made me really sad because it was quite a nice one and it just encapsulated the state of our marriage generally—some torn up shreds of paper featuring a torn up Marmite jar. (Josie)

There were many examples of “Amy” being resistant to adults around her, and Josie explained, “You need to be made of strong stuff,” and events quickly escalated, whereby it was “almost like lighting a piece of paper on fire. You know one minute you’ve got a piece of paper, the next minute, you’ve got a fire” (Josie).

## Discussion

The initial framing of the phenomenon of non-intentional filial harm as “explosive and controlling impulses” had connotations when reflecting on how these are individually used in the broader field of family violence. Control, as a term, is often conflated with coercive control (Stark, 2007), controlling behavior (Stark & Hester, 2019), and sits on the continuum of sexual violence (Kelly, 1987). Coercive control exists as a deliberate pattern of incidents with the purpose of creating an environment where a “victim” adapts their behavior and performs roles of responsibilities desired by the “perpetrator” (Stark, 2007; Stark & Hester, 2019), and The Serious Crime Act 2015 defines controlling behavior as:

a range of acts designed to make a person subordinate and/or dependent by isolating them from sources of support, exploiting their resources and capacities for personal gain, depriving them of the means needed for independence, resistance and escape and regulating their everyday behaviour.

This contradicts what parents shared above and how children described their behaviors and impulses, which were about meeting a need and not designed to deliberately manipulate others or negotiate the power relationship. Furthermore, in the example given by “Katie” above, although describing some harms as controlling would not stop her from accessing support, it did evoke discomfort. Instead, when adapting our co-produced “explosive and controlling impulses” for broader use, “harmful” is more appropriate than “controlling.” An example of this is its use in “harmful sexual behaviors,” which are defined as:

Sexual behaviour expressed by children and adolescents under the age of 18 which is developmentally inappropriate, may be harmful towards the child or adolescent themselves or others, or be abusive towards another child, adolescent or adult. (Hackett et al., 2016, np)

Here the caveat was the “abusive” behavior must have a coercive element and/or a power dynamic, which was specifically not relevant to children who did not *intend* to cause harm.

Furthermore, definitions using “harm” rather than control are particularly relevant to this research, as when children and young people engage in harmful behaviors, they frequently also cause themselves significant harm. Considering harmful behaviors to be “developmentally inappropriate” and “harmful toward the child or adolescents themselves or others” means it is better aligned to unintentional forms of harm than the terminology of control, in line with what co-researchers reflected upon. One of the limitations of this research is how parent co-researchers worked with me on an individual level, and child co-researcher groups were siloed. For broader reflection and discussion, future research should consider bringing co-researchers together to discuss the research processes and plans in much further detail.

Explosive and harmful impulses are the overarching Grounded Theory of this research, with the PRAR framework representing the nuance between the different needs, and the different impulses experienced by families where there is filial harm. However, a clear limitation of this research is lack of descriptive detail that could help families identify which need is being represented where there is filial harm as, as mentioned, the harm may look identical despite there being distinct and different needs being presented. Glaserian Grounded Theory often fails to describe the phenomena of interest effectively, as it focuses so heavily on the conceptual (Rutter, 2021). As anxiety, hyperactivity, and sensory differences were all identified as features which outline the distinct differences between the different subcategories, this supports previous arguments that neurological assessments may be useful for children where there is filial harm (Rutter, 2021).

Utilizing the language of explosive and harmful impulses rather than violence, abuse, or aggression has the potential to open referral pathways which feel more relevant to those families seeking support for such harms. Thus, moving such harms away from being considered “hidden” and moving toward an open conversation regarding the support needs of parents, children, and the wider family system. It does not apply blame, which was one of the leading concerns of parents in this research, but rather opens a dialogue around the interpretation of needs and the strategies that are or are not working, within the home already. This is also true of those researching within the field of filial harm, whereby the broader language of filial harm or the wide variety of terms identified in the introduction of this article can be used as umbrella terminology, whereas explosive and harmful impulses are specifically relating to non-intentional forms of harm. This expands recognition into the different experiences of families; mothers appear to be seeking support for this phenomenon more than fathers (Rutter, 2023), as well recognizing as the diversity of those living with this form of harm which could potentially expand further exploration of filial harm and unmet mental health needs, neurodivergence, and age of both children and their parents (Rutter, 2021, 2022, 2023).

## Conclusion

Explosive and harmful impulses provide a description of filial harms, which is specifically related to non-intentional harms caused by children when they are attempting to meet other needs. Using the “PRAR” framework of trying to understand and interpret these harms as a method of meeting needs proactively, reactively, affectively, or relationally has significant potential regarding practitioner approaches and the direction of future research into harms where “intent to harm” does not appear to be the main driver. Thus, it is recommended providers of interventions for filial harm instigated by pre-adolescents utilize the language of “explosive and harmful impulses” and approach the phenomenon as a needs-based issue. This approach, language, and description could represent not only the experiences of preadolescent children and their families, such was the case in this research, but there is opportunity to extend the concept of “explosive and harmful impulses” and the PRAR work to neurodivergent youth, as the majority of the parents in this research identified their children as neurodivergent, particularly through the lens of PDA, and many filial harms instigated by neurodivergent youth could be considered non-intentional.

As to what is the most appropriate umbrella term to attempt to capture all forms of harm to a parent-figure instigated by their pre-adolescent, adolescent, or adult child continues to be debated. However, such expansive terminology loses the nuance, source, and purpose of these harms. Umbrella terminology is useful at the political and policy-based level; it can create pathways to funding and information, but exploring explosive and harmful impulses as a subset of these harms also provide opportunity to work with families using the language *they* helped develop, that speaks to them and their experiences. Thus, when potential interventions, support pathways, and research with families living with explosive and harmful impulses are developed, the PRAR framework should be considered to understand why children are attempting to meet their needs in harmful ways and then explore how they could be provided with less harmful, more appropriate methods to meet those underlying needs.
